# *COX-2 *expression in papillary thyroid carcinoma (PTC) in cytological material obtained by fine needle aspiration biopsy (FNAB)

**DOI:** 10.1186/1756-6614-4-3

**Published:** 2011-01-10

**Authors:** Kinga Krawczyk-Rusiecka, Katarzyna Wojciechowska-Durczyńska, Anna Cyniak-Magierska, Zbigniew Adamczewski, Elżbieta Gałecka, Andrzej Lewiński

**Affiliations:** 1 Department of Endocrinology and Metabolic Diseases, Medical University of Lodz, Polish Mother's Memorial Hospital - Research Institute, Lodz, Poland

## Abstract

**Background:**

COX-2 is an enzyme isoform that catalyses the formation of prostanoids from arachidonic acid. An increased *COX-2 *gene expression is believed to participate in carcinogenesis. Recent studies have shown that *COX-2 *up-regulation is associated with the development of numerous neoplasms, including skin, colorectal, breast, lung, stomach, pancreas and liver cancers. *COX-2 *products stimulate endothelial cell proliferation and their overexpression has been demonstrated to be involved in the mechanism of decreased resistance to apoptosis. Suppressed angiogenesis was found in experimental animal studies as a consequence of null mutation of *COX-2 *gene in mice. Despite the role of *COX-2 *expression remains a subject of numerous studies, its participation in carcinogenesis or the thyroid cancer progression remains unclear.

**Methods:**

Twenty three (23) patients with cytological diagnosis of PTC were evaluated. After FNAB examination, the needle was washed out with a lysis buffer and the obtained material was used for *COX-2 *expression estimation. Total RNA was isolated (RNeasy Micro Kit), and RT reactions were performed. β-actin was used as endogenous control. Relative *COX-2 *expression was assessed in real-time PCR reactions by an ABI PRISM 7500 Sequence Detection System, using the ΔΔC_T _method.

**Results:**

*COX-2 *gene expression was higher in patients with PTC, when compared to specimens from patients with non-toxic nodular goitre (NTG).

**Conclusions:**

The preliminary results may indicate *COX-2 *role in thyroid cancer pathogenesis, however the observed variability in results among particular subjects requires additional clinical data and tumor progression analysis.

## Background

The most common thyroid malignancy is papillary thyroid carcinoma (PTC), accounting for approximately 85-90% of all thyroid cancers. PTC usually grows slowly and is clinically indolent, although aggressive forms can also occur. The 10-year survival rate for all PTC patients is estimated at 80-90% [1].

The introduction of fine-needle aspiration biopsy (FNAB) has made PTC identification more accurate, but still the diagnostic tools regarding the differentiation between thyroid benign and malignant lesions are not always reliable. The molecular basis of PTC has been examined and association with variety of molecular prognostic markers has been described, including *RAS, RET, Trk, MET*, and *BRAF *mutations [2].

Cyclooxygenase-2 (COX-2) is an enzyme isoform involved in the conversion of arachidonic acid to prostaglandin H_2_, the precursor of various molecules, including prostaglandins, prostacyclines and thromboxanes. COX-2 can be expressed in response to various stimuli, such as hormones, mitogens, cytokines, inflammatory mediators and growth factors *via *protein kinase C and RAS-mediated signaling [3,4]. The products of COX-2 activity are believed to be involved in carcinogenesis by promoting angiogenesis, inhibiting apoptosis, increasing cell invasion and stimulating cell proliferation. COX-2 also modulates vascular endothelial growth factor (VEGF) production, the factor that promotes angiogenesis and decreases immunity toward cancer cells [5-9].

Recent studies have highlighted the relevance of COX-2 in human carcinogenesis. Increased levels of COX-2 have been reported in numerous tumours, including head and neck squamous cell cancer, as well as colorectal, breast, lung, skin, stomach, liver, pancreas, bladder, ovaries and prostate carcinomas [10-19].

Selective inhibition of COX-2 is a novel therapy under investigation in both the chemoprevention and treatment of solid tumors [20,21]. Experimental animal data indicate that *COX-2 *gene may be associated with carcinogenesis of several types of human malignancies.

Whether *COX-2 *expression is related to the morphology of PTC remains unclear. In the present study we have investigated *COX-2 *expression in 23 cases of PTC diagnosed cytologically following FNAB.

## Methods

45 thyroid specimens were analyzed, i.e. 23 cases of PTC and 22 cases of benign thyroid lesions; 6 men and 39 women participated in the study. Patients ranged in age from 24 to 87 years (median - 48 years in PTC group, 58 years in benign thyroid lesions group). Thyroid aspirates, eligible for the study, were obtained from patients by fine needle aspiration biopsy (FNAB) in the Clinical Hospital No. 3, Medical University of Lodz (2007-2010). Each aspirate was smeared for conventional cytology, while the remaining part of aspirate was immediately washed out of the needle. The cells, obtained from the needle, were used in further investigation. Macroscopically unchanged thyroid tissue, collected from patients treated surgically, was used as an internal control.

The procedures, used in the study, were approved by the Ethical Committee of the Medical University of Lodz (Poland). All patients were informed and agreed to participate in this study.

Total RNA was extracted using RNeasy Micro Kit (Qiagen, Hilden, Germany), based on modified Chomczyński and Sacchi's method [22]. RNA concentration was spectrophotometrically assessed by measuring absorbance at 260 and 280 nm (NanoDrop ND-1000 Spectrophotometer, Thermo Fisher Scientific, USA).

Total RNA was reversely transcribed in a Mastercycler personal thermocycler (Eppendorf, Hamburg, Germany), according to manufacturer's procedures in a total volume of 50 μl including oligo d(T)16 (50 μM), MultiScribe Reverse Transcriptase (50 U/μl), 10 × TaqMan RT Buffer, MgCl_2 _solution (25 mM), dNTPs mixture (10 mM), RNAse Inhibitor (20 U/μl) and nuclease-free water (TaqMan Reverse Trancriptase Reagents, Applied Biosystems, CA, USA). The reactions were incubated for 10 min in 25°C, 30 min in 48°C, heated for 5 min in 95°C and placed at 4°C.

The relative expression of *COX-2 *gene was assessed, using the ABI PRISM 7500 SDS Software (ABI PRISM 7500 Sequence Detection System, Applied Biosystems), according to the manufacturer's protocol. The PCR reactions for COX-2 gene were run with 5 μl of cDNA in a total volume of 50 μl, using TaqMan Universal PCR Master Mix (Applied Biosystems, Foster City, CA, USA) and predesignated primer/probe set (Assays-on-Demand™ Gene Expression assay, Hs 00153133_m1, Applied Biosystems). Amplification reactions were done in triplicate for each examined sample.

Controls with no template cDNA were used with each assay. The reference gene was β-actin (Assays-on-Demand™ Gene Expression assay, Hs 99999903_m1, Applied Biosystems). Samples of cDNA were quantified using a fluorescence based real-time detection method (TaqMan).

Assays-on-Demand Gene Expression product consists of 20 × mix of unlabelled PCR primers and TaqMan MGB probe (5 μM) with FAM™(6-carboxy-fluorescein) at the 5' end as the reporter dye and a non-fluorescent quencher (TAMRA, 6-carboxy-tetramethylrhodamine) at the 3' end. Cycling conditions were 50°C for 2 min and 95°C for 10 minutes, followed by 50 cycles at 95°C and 60°C for 1 minute. A sample of normal thyroid tissue served as a calibrator to compare the relative amount of target in different samples and to adjust for the plate-to-plate variation in amplification efficiency.

### Data and statistical analysis

Relative gene expression - determined by comparing threshold cycle (C*_T_*) for gene of interest (COX-2) with C_*T*_for the reference gene (β-actin) - was calculated using the ΔΔC_*T*_method as described previously [23]. The characteristics, RQ and ΔC_*T*_values of patients groups are shown in Table 1 and Table 2.

**Table 1:** Gender, age, ddCT and RQ values in PTC group.

No.	Gender	Age	ddCT	RQ
1.	M	56	-0.84	1.79

2.	M	64	-0.71	1.63

3.	F	52	-6.49	89.8

4.	F	28	0.95	0.52

5.	F	53	-1.62	3.06

6.	M	61	-0.62	1.53

7.	F	57	-2.46	5.49

8.	F	36	-4.07	16.74

9.	F	56	-2.76	6.77

10.	M	58	-3.12	8.69

11.	F	28	1.31	0.4

12.	F	24	-1.31	2.48

13.	F	67	-1.51	2.84

14.	F	no data	1.24	2.36

15.	F	39	-2.0	4.01

16.	F	49	-0.75	1.68

17.	F	28	2.86	0.14

18.	F	45	-2.75	6.73

19.	F	50	-2.11	4.31

20.	F	63	-0.86	1.82

21.	M	53	-3.98	15.8

22.	F	39	-3.44	10.84

23.	F	49	-2.22	4.66

**Table 2:** Gender, age, ddCT and RQ values in NTG group.

No.	Gender	Age	ddCT	RQ
1.	F	27	-0.84	1.79

2.	F	87	-0.41	1.32

3.	F	65	1.98	0.25

4.	F	72	-1.01	2.02

5.	F	64	-2.32	4.71

6.	F	68	-0.01	1.01

7.	F	46	-2.07	4.21

8.	F	39	1.55	0.34

9.	M	56	-0.63	1.55

10.	F	68	-0.98	1.98

11.	F	62	2.94	0.13

12.	F	48	1.96	0.26

13.	F	32	-2.91	7.51

14.	M	61	-0.21	1.15

15.	F	29	0.74	0.6

16.	F	88	-0.68	1.6

17.	M	71	-1.53	2.88

18.	F	43	-0.46	1.37

19.	F	54	0.77	0.24

20.	F	50	-1.2	2.3

21.	F	79	-1.37	2.59

22.	F	71	-0.93	1.9

Statistical analysis was performed, using a standard parametric Student's t test, followed by non-parametric U Mann-Whitney's test. P values < 0.05 were considered to indicate statistical significance.

## Results

**Figure 1:**
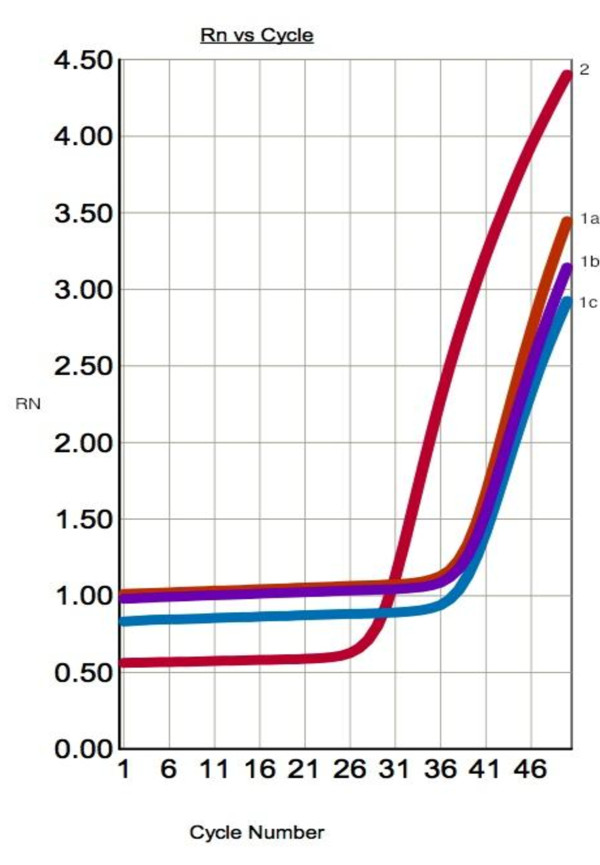
**Representative amplification curves for *COX-2 *and *β-actin *genes in real-time PCR in PTC specimen**. 1 a,b,c - amplification curve for *COX-2 *gene (mean *C*_*T *_value *- 36.694); *2 - amplification curve for *β-actin *gene (endogenous control) (mean C*_T_* value - 27.071).

**Figure 2:**
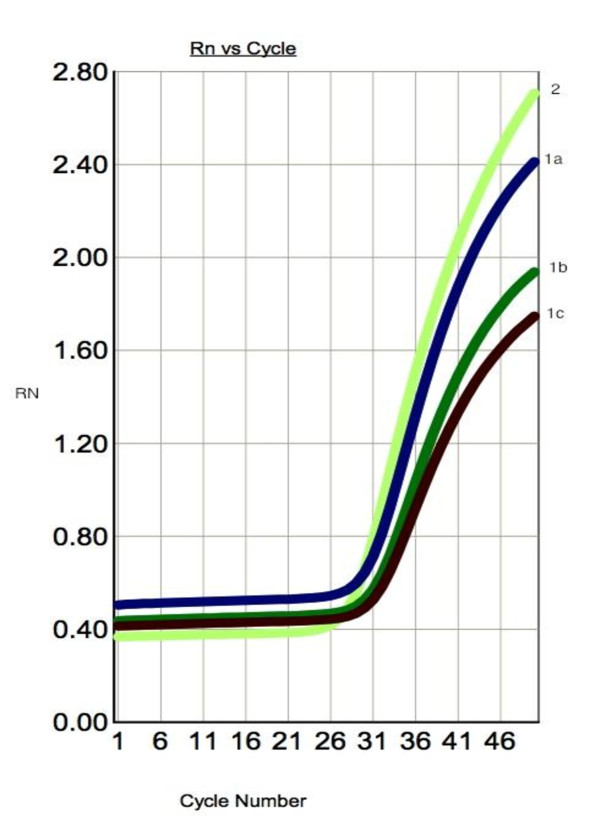
**Representative amplification curves for *COX-2 *and *β-actin *genes in real-time PCR in macroscopically unchanged specimen**. 1 a,b,c - amplification curve for *COX-2 *gene (mean *C*_*T *_value *- 31.979); 2 - a*mplification curve for *β-actin gene *(endogenous control) (mean *C_T_* value - 27.250).

**Figure 3:**
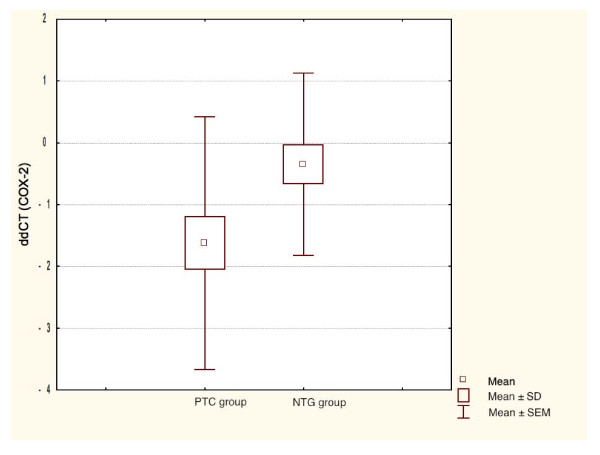
**Box-and-whisker plots representing the COX-2 mRNA expression by quantitative RT-PCR in PTC and NTG groups**. Results are calculated as ddCT values. Whiskers represent means ± SD (standard deviation) for particular groups. Boxes represent means ± SEM (standard error of mean). The results were statistically analyzed, using Student's t test.

**Figure 4:**
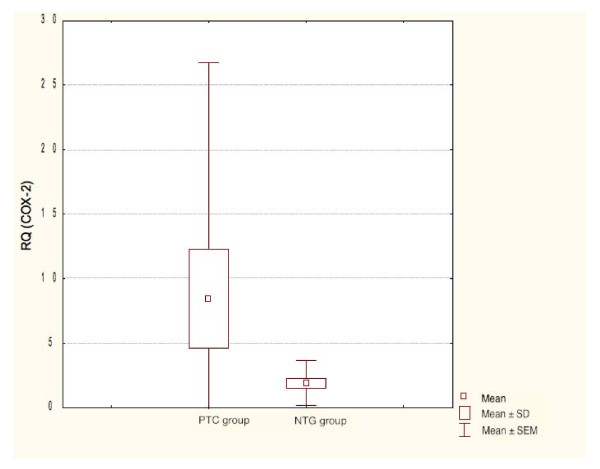
**Box-and-whisker plots representing the COX-2 mRNA expression by quantitative RT-PCR in PTC and NTG groups**. Results are calculated as RQ values. Whiskers represent means ± SD (standard deviation) for particular groups. Boxes represent means ± SEM (standard error of mean). The results were statistically analyzed, using Man-Whitney's U test (p < 0.05).

COX-2 mRNA expression was significantly higher in PTC when compared to benign thyroid lesions (Student's t-test, P = 0.021). There was no significant relationship between *COX-2 *expression and patients age and sex. The amplification plots of *COX-2 *and *β-actin *are shown in Figure. 1 and 2. The statistical analysis of *COX-2 *expression by quantitative Real Time PCR in PTC and NTG is presented in Figure. 3 and Figure. 4.

## Discussion

The relation between *COX-2 *activity and carcinogenesis is being examined in variety of human neoplasms. Fine needle aspiration biopsy has become a critical diagnostic tool in management of thyroid nodules. It has allowed to decrease the number of cases inquiring surgical treatment from 67% to 44%; also the percentage of operated carcinomas in those nodules has increased from 14% to 29% [24]. Generally, preoperative discrimination of the thyroid malignancy, using not only cytopathology but also molecular markers, would enhance the proper diagnosis.

In our study, COX-2 expression in both NTG and PTC were examined. *COX-2 *expression was significantly higher in PTC group.

All but one of the previously conducted studies found overexpression of COX-2 in cases of PTC and follicular thyroid carcinoma (FTC). In the study of Ito et al. [25], there were 9 COX-2-negative (18,4%) among 49 of PTC cases, studied by immunochemistry. They also suggested significant reduction of COX-2 levels in older patients (over 54 years old), in patients with large tumors and with the advanced disease stages, as well as with the presence of solid, scirrhous or trabecular growth pattern. An association among and age and worse prognosis has been well-recognized in the medical literature. Accordingly, Ito et al. [25] found that the COX-2 levels were significantly reduced in older patients, whereas Siironen et al. [26] demonstrated the older age group of patients, having a much worse prognosis and higher COX-2 levels. However, in our study, we have failed to establish statistical significance between COX-2 expression and patients' age or sex which is similar to results of Garcia-Gonzales et al [27].

The results of Kajita et al. [28] did not show any significant differences in COX-2 expression between normal thyroid tissue and PTC, because of variation in mRNA levels. The authors have also performed the *in vitro *study, with the TPC-1 thyroid carcinoma cell line and a compound NS-398 - a COX-2 enzymatic activity specific inhibitor, showing inhibited growth of tumor cells. They concluded the role of COX-2 in the growth of PTC cell lines.

Cornetta et al. [29] examined a variety of thyroid tissue specimens; COX-2 staining was not observed in none of the 6 specimens obtained from normal or multinodular goiter specimens. Analysis of Hashimoto's thyroiditis revealed COX-2 expression in the follicular epithelium and lymphocytic infiltrates, as well as in cases of FTC and PTC [29].

The study of Lee et al. [30] showed prominent expression of COX-2 in thyroiditis, benign and malignant thyroid lesions but not in normal thyroid tissues. They also observed no correlation between severity of PTC, regardless of the presence or absence of metastases. Because of the same intensity of COX-2 staining, found in thyroiditis, benign and malignant thyroid lesions the authors concluded that it is unlikely that COX-2 expression is related to the progression of thyroid disease.

The results of study with *Apc*^Δ716 ^knockout mice [31], suggested that the induction of *COX-2 *was a very early event in colon carcinogenesis. This view has been supported by Garcia-Gonzales et al. [28] who have suggested that even though COX-2 plays an important role in progression of all thyroid cancers, in case of PTC it seems to be more important only in the early stages of disease.

## Conclusions

The usefulness of *COX-2 *as a marker of thyroid malignancy has been challenged but its potential role in carcinogenesis arouses significant interest. In the present study, *COX-2 *expression was found in all studied samples and the elevated level of *COX-2 *gene expression was confirmed in PTC genetic material, collected from the fine-needle washout fluids. Thus, *COX-2 *gene may play a significant role in the pathogenesis of this cancer type. Furthermore, *COX-2 *gene overexpression levels were individually diversified, what may have resulted from their association with different genetic and clinical prognostic factors.

## Competing interests

The authors declare that they have no competing interests.

## Authors' contributions

KK-R designed and coordinated the study, carried out the molecular genetic studies and drafted the manuscript. KW-D participated in performing molecular studies. AC-M participated in performing molecular studies. ZA and EG participated in coordination of molecular genetic studies. AL, the senior author, wrote the manuscript. All authors read and approved the final manuscript.
